# Electroconvulsive Therapy (ECT): A Scotland Wide Naturalistic Study of 4,826 treatment episodes

**DOI:** 10.1192/j.eurpsy.2024.223

**Published:** 2024-08-27

**Authors:** J. Langan Martin, M. Fleming, D. Varveris, S. Kelly, D. Martin

**Affiliations:** ^1^University of Glasgow; ^2^NHS Greater Glasgow and Clyde; ^3^NHS Forth Valley, Glasgow, United Kingdom

## Abstract

**Introduction:**

Despite its apparent efficacy in the treatment of a range of psychiatric disorders, electroconvulsive therapy (ECT) is viewed by some as a contentious treatment. Although most clinicians and researchers consider ECT a safe and effective treatment, there are ongoing and significantly publicised concerns about potential side effects.

**Objectives:**

To explore use of ECT across Scotland in a large naturalistic clinical sample across an 11-year period from 2009 to 2019. To consider the efficacy and side effects of ECT for a range of common psychiatric disorders including, depression, bipolar depression, schizophrenia, and mania.

**Methods:**

Using data from the Scottish Electroconvulsive Therapy (ECT) Accreditation Network (SEAN), information was collected for all adults who had received ECT. Variables included age, sex, Scottish Index of Multiple Deprivation (SIMD) quintile, International Classification of Diseases, Tenth Edition (ICD-10) diagnosis, indication for ECT, Mental Health Act status, consent status, entry and exit Montgomery-Asberg Depression Rating Scores (MADRS), entry and exit Clinical Global Index Severity CGI-S) scores and reported side effects. Side effects were recorded as present if the side effect was reported at any point during the episode of treatment.

**Results:**

4826 ECT episodes were recorded. The majority of episodes were in women (68.4%, n=3,301). Average age at treatment onset was 58.52 years. Males were slightly younger (m=58.24 years vs f= 58.65 years, p= 0.20). Mean number of treatments/episode was 9.59 (95% CI 9.32 – 9.85). Mean treatment dose delivered was 277.75mC (95%CI 272.88 – 282.63mC).

2920 episodes of treatment had CGI-S entry and exit recorded. At entry, mean CGI-S indicated marked illness (5.03 95% CI 4.99-5.07). Recipients with schizophrenia had the highest CGI-S score (5.45 95% CI 5.21-5.60), followed by those with post-partum disorders (5.38, 95% CI 4.61-6.14). At exit, mean CGI scores indicated borderline illness (2.07, 95% CI 2.03-2.11), recipients diagnosed with mixed affective state had the lowest CGI-S score (1.72, 95% CI 0.99-2.47) followed by those with schizoaffective disorder (2.01, 95% CI 1.76-2.42).

Anaesthetic complications (n=34) and prolonged seizures (n=38) were rare, occurring in <1% of treatment episodes. Cardiovascular complications were reported in 2.2% (n= 102). Nausea was reported in 7.2% (n= 334) and muscle aches in 12% (n=560). Confusion was reported in 19% (n=879) and cognitive side effects were reported in 26.2% (n=1212). One third of treatment episodes reported confusion or cognitive side effects (33.1%, n=1545).

**Conclusions:**

From this large naturalistic clinical sample, ECT appears to be effective in improving illness severity as measured by CGI-S score. While some side effects (such as prolonged seizures and cardiovascular complications) were rare, others (such as confusion or cognitive side effects) were relatively common.

**Disclosure of Interest:**

None Declared

